# The gamma chain subunit of Fc receptors is required for alpha-synuclein-induced pro-inflammatory signaling in microglia

**DOI:** 10.1186/1742-2094-9-259

**Published:** 2012-11-27

**Authors:** Shuwen Cao, David G Standaert, Ashley S Harms

**Affiliations:** 1 Center for Neurodegeneration and Experimental Therapeutics, Department of Neurology, The University of Alabama at Birmingham, 1719 6th Ave. South, CIRC 516, Birmingham, AL, 35294-0021, USA

**Keywords:** Neuroinflammation, FcγR, NF-κB, Microglia

## Abstract

**Background:**

The protein alpha-synuclein (α-SYN), which is found in the Lewy bodies of dopamine-producing (DA) neurons in the substantia nigra (SN), has an important role in the pathogenesis of Parkinson’s disease (PD). Previous studies have shown that neuroinflammation plays a key role in PD pathogenesis. In an AAV-synuclein mouse model of PD, we have found that over-abundance of α-SYN triggers the expression of NF-κB p65, and leads to microglial activation and DA neurodegeneration. We also have observed that Fcγ receptors (FcγR), proteins present on the surface of microglia that bind immunoglobulin G (IgG) and other ligands, are key modulators of α-SYN-induced neurodegeneration.

**Methods:**

In order to study the role of FcγRs in the interactions of α-SYN and microglia, we treated the primary microglial cultures from wild-type (WT) and FcγR^−/−^ mice with aggregated human α-SYN *in vitro*.

**Results:**

Using immunocytochemistry, we found that α-SYN was taken up by both WT and FcγR^−/−^ microglia, however, their patterns of internalization were different, with aggregation in autophagosomes in WT cells and more diffuse localization in FcγR^−/−^ microglia. In WT microglia, α-SYN induced the nuclear accumulation of NF-κB p65 protein and downstream chemokine expression while in FcγR^−/−^ mouse microglia, α-SYN failed to trigger the enhancement of nuclear NF-κB p65, and the pro-inflammatory signaling was reduced.

**Conclusions:**

Our results suggest that α-SYN can interact directly with microglia and can be internalized and trafficked to autophagosomes. FcγRs mediate this interaction, and in the absence of the gamma chain, there is altered intracellular trafficking and attenuation of pro-inflammatory NF-κB signaling. Therefore, blocking either FcγR signaling or downstream NF-κB activation may be viable therapeutic strategies in PD.

## Background

Parkinson’s disease (PD) is a degenerative neurological disorder characterized primarily by loss of dopaminergic (DA) neurons from the substantia nigra pars compacta (SNpc). Genetic and biochemical evidences have established a close link between the protein alpha-synuclein (α-SYN) and the pathogenesis of the disease [1,2]. The disease may be triggered by mutations or overexpression of α-SYN, and all cases of PD are associated with accumulation of insoluble α-SYN [2,3]. The most prominent neuropathological features, intraneuronal Lewy bodies and Lewy neurites, are composed mainly of fibrillar α-SYN [1]. However, the mechanism by which excess and modified α-SYN leads to the degenerative process in PD is still unclear.

Neuroinflammation plays an important role in the pathogenesis and progression of PD. Microglial activation and T-lymphocyte infiltration are consistently observed in the SN of PD patients and in the animal models of PD [4-6]. Meanwhile, cytokines like tumor necrosis factor alpha (TNF-α), interleukin 1-beta (IL-1b), and interleukin 6 (IL-6) show increased concentration in the serum and cerebrospinal fluid of PD patients [7]. Moreover, a genome-wide association study has shown that polymorphisms in HLA-DR are associated with sporadic PD [8-10]. In previous studies, we have used a mouse model in which α-SYN is overexpressed using an adeno-associated viral vector (AAV) to reproduce many of these features, including IgG deposition, classical microglial activation with increased production of pro-inflammatory cytokines, and B- and T-lymphocyte infiltration in the SN [11]. The prominence of this inflammatory response to α-SYN overexpression has led us to explore the mechanisms responsible for α-SYN-induced immune activation.

Fc gamma receptors (FcγR) are proteins expressed on the surface of microglia as well as other cell types, including natural killer cells, neutrophils, and mast cells. They bind to immunoglobulin G (IgG) and some non-IgG ligands, such as complement receptors and C-reactive proteins [12-14], and can trigger microglial activation and cellular responses. In our previous studies *in vivo*, we have found that the Fc receptors appear to have a key role in α-SYN-induced inflammation: deficiency of FcγRs blocks α-SYN-induced NF-κB-driven pro-inflammatory signaling, and attenuates microglial activation and DA neurodegeneration [15].

In order to study whether excess α-SYN interacts directly with microglia and what role FcγRs play in this process, we treated the primary microglia of wild-type (WT) and FcγR^−/−^ mice with human α-SYN *in vitro*. We found that microglia can internalize aggregated α-SYN, and this leads to activation of NF-κB signaling with downstream induction of chemokines. This process is modulated by FcγRs, even in the absence of IgG. These data show that FcγRs play a role in the interaction of microglia with aggregated α-SYN, and targeting these interactions may be useful in modifying the inflammatory state in PD.

## Methods

### Animals

C57BL/6 mice and FcγR^−/−^ mice were used for the study. The FcγR^−/−^ mice in a C57BL/6 background were obtained from Taconic labs (model # 000583-M-M, Taconic, Hudson, NY, USA). These mice (nomenclature: B6.129P2-Fcer1g^tm1Rav^N12) are deficient in the gamma chain subunit which is found in several members of the Fc family: FcγRI, FcγRIII, and FcεRI. They exhibit immune system defects such as inability to phagocytose antibody-coated particles, and the inflammatory responses to immune complexes are attenuated [16]. All experiments were carried out in compliance with the USPHS Guide for Care and Use of Laboratory Animals. All experiments were approved by the Institutional Animal Care and Use Committee (IACUC) of The University of Alabama at Birmingham with Animal Protocol Number 100908919.

### Mouse primary microglia culture and α-SYN treatment

Microglia were isolated from postnatal day 0 to 3 (P0-P3) C57BL/6 mice and FcγR^−/−^ mouse pups according to published protocols [17] with minor modifications. In brief, whole brains were isolated, minced, and placed in ice-cold dissociation media containing sterile filtered DNase1 (1 μL/mL, Invitrogen, Carlsbad, CA, USA), Dispase II (1.2 U/mL, Roche, Indianapolis, IN, USA), and Papain (1 mg/mL, Sigma-Aldrich, St. Louis, MO, USA) dissolved in DMEM/F12 (Sigma-Aldrich, St. Louis, MO, USA). Cells were dissociated for 10 min at 37°C with agitation every few minutes. After mechanical and chemical dissociation, the population of mixed glial cells was filtered through a 40 μm-pore filter (BD Falcon, Franklin Lakes, NJ, USA) and plated on T75 flasks in DMEM/F12 supplemented with 20% heat-inactivated fetal bovine serum (FBS, Sigma-Aldrich, St. Louis, MO, USA), 1% penicillin/streptomycin (Sigma-Aldrich, St. Louis, MO, USA), and 1% L-glutamine (Sigma-Aldrich, St. Louis, MO, USA). Mixed glial cultures were maintained in culture in a humidified incubator at 37°C and 5% CO_2_ for 14 to 16 days and media were replenished every 3 to 4 days. Once cultures reached confluence, primary microglial cells were isolated from the astroglial cell bed by mechanical agitation on an orbital shaker (150 rpm) for 1 h at 37°C. After isolation, cells were plated in DMEM/F12 supplemented with 1% penicillin/streptomycin and 1% L-glutamine at a density of 70,000 cells/well in a four-well chamber slide (LAB-TEK, Rochester, NY, USA) for immunocytochemistry, ELISA, and multiplex assay.

Purified human α-SYN (1 mg/mL, r-Peptide, Athens, GA, USA) was incubated at 37°C with agitation for 7 days as previously described [18], and pulse sonicated for 2 s before adding into the primary microglia culture. In order to determine the aggregated state of the α-SYN used in these experiments, aliquots of the α-SYN preparation were separated on Superdex Column into 1 mL fractions. All fractions were analyzed by western using a monoclonal antibody (LB509, Abcam, Cambridge, MA, USA) for human α-SYN. Western analysis indicated aggregates of approximately 1 MDa (Additional file 1: Figure S1). The primary microglia were treated with 500 nM aggregated human α-SYN at different time points.

### Immunocytochemistry

Twenty-four, 48, and 72 h after α-SYN treatment, anti-CD45 and anti-human α-SYN antibodies were used to study α-SYN internalization and localization. For examining NF-κB activation, we used anti-NF-κB p65 antibody and SYTOX Green nucleic acid stain for primary microglia 24 h and 72 h post treatment.

Cells were fixed with 4% paraformaldehyde, permeabilized with TBS containing 3% gelatin from cold water fish skin (Sigma-Aldrich, St. Louis, MO, USA), 1% BSA, and 0.5% Triton X-100, blocked with TBS containing 3% gelatin from cold water fish skin and 1% BSA. Primary antibody incubations were done for 2 h at room temperature with primary antibodies diluted in TBS containing antibody diluent (TBS containing 3% gelatin from cold water fish skin, 1% BSA, and 0.1% Triton X-100), rat anti-CD45 (1:500, AbD Serotec, Kidlington, UK), mouse anti-human α-SYN (1:500, Abcam, Cambridge, MA, USA), rabbit anti-human α-SYN (1:500, Cell Signaling Technology, Danvers, MA, USA), rabbit anti-LC3B (1:200, Abcam, Cambridge, MA, USA), rat anti-LAMP-1 (1:100, DSHB at the University of Iowa, Iowa City, IA, USA), or goat anti-NF-κB p65 (1:100, Santa Cruz Biotechnology, Santa Cruz, CA, USA) followed by a 1:500 dilution of alexa-488 conjugated goat anti-rabbit, goat anti-mouse, donkey anti-rat (Molecular probes, Eugene, OR, USA), a 1:500 dilution of CY3-conjugated goat anti-rat, goat anti-rabbit, donkey anti-rabbit, or donkey anti-goat (Jackson Immunoresearch, West Grove, PA, USA) antibodies and 0.05 μM SYTOX Green nucleic acid stain (Invitrogen, Carlsbad, CA, USA). Each experimental set was repeated two to three times.

### Imaging and quantification

Confocal images were captured using a Leica TCS-SP5 laser scanning confocal microscope. The images were processed using the Leica software and exported as TIFF files and processed using Adobe Photoshop CS2. For quantitation of NF-κB p65 staining, the nuclear regions of the cells were defined using SYTOX Green staining, and the p65 intensity was determined using region of interest analysis with ImageJ software ( http://rsbweb.nih.gov/ij/). Intensity scores obtained from four images per group (5 to 15 cells in each image) were statistically analyzed using *t* test.

### ELISA

Conditioned media were collected 2, 4, 8, and 16 h after the treatment of primary microglia with vehicle or aggregated human α-SYN. The quantities of MIP-1α were measured with a mouse MIP-1α ELISA kit (R&D Systems, Minneapolis, MN, USA) per the manufacturer’s instructions. The quantities of TNF were measured with a mouse TNF ELISA kit (eBiosciences, San Diego, CA, USA) per manufacturer’s instructions.

### Multiplex assay

Conditioned media were collected 4 h and 24 h after the treatment of primary microglia with vehicle or aggregated human α-SYN for each of three independent experiments, and analyzed for mouse cytokine and chemokine production on an assay panel with 25 analytes (G-CSF, GM-CSF, IFN-γ, IL-10, IL-12 (p40), IL-12 (p70), IL-13, IL-15, IL-17, IL-1α, IL-1β, IL-2, IL-4, IL-5, IL-6, IL-7, IL-9, IP-10, KC-like, MCP-1, MIP-1α, MIP-1β, MIP-2, RANTES, TNF-α) per the manufacturer’s instructions (Millipore, Billerica, MA, USA).

## Results

### Internalization of aggregated human α-SYN by mouse primary microglia

**Figure 1:**
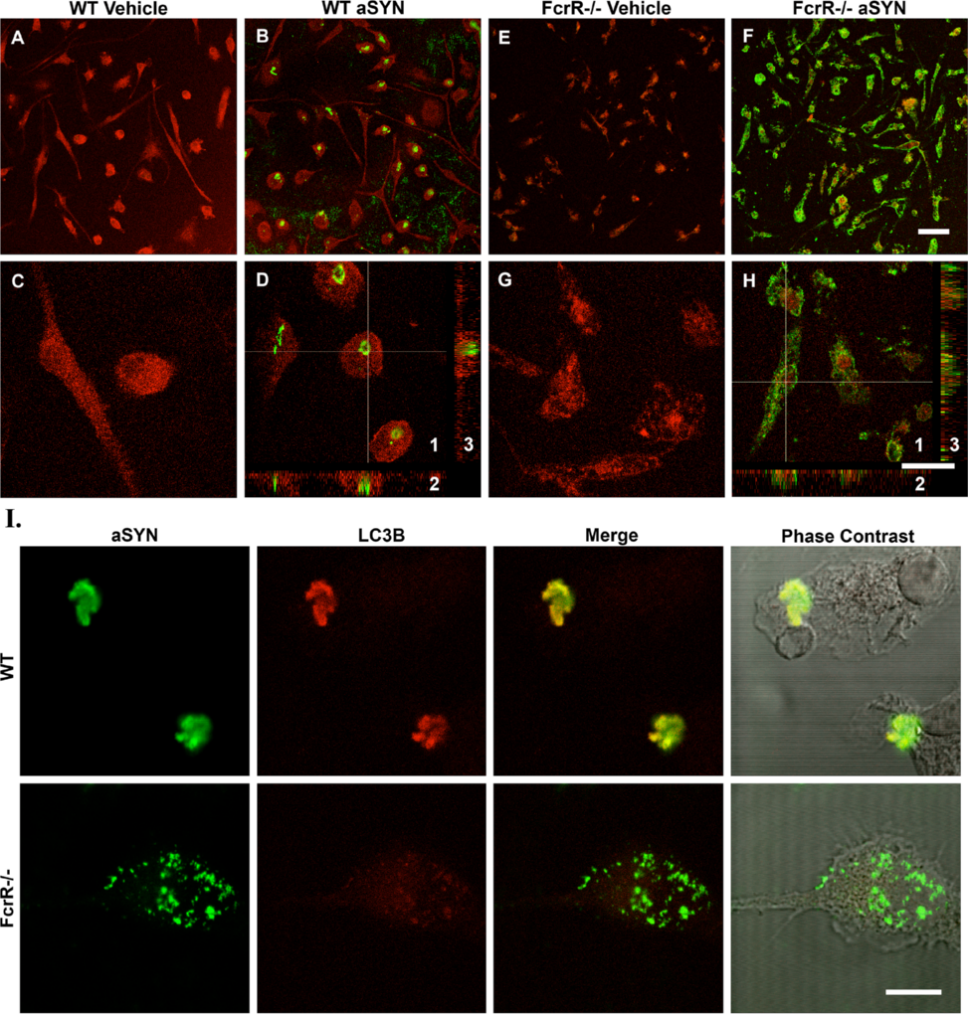
**α-SYN internalization in WT and FcγR**^**−/−**^**mouse primary microglia.** (**A-H**) 72 h after the treatment of aggregated human α-SYN, WT and FcγR^−/−^ mouse primary microglia were immunostained for human α-SYN (Green) and the microglial marker CD45 (Red). Condensed α-SYN was observed in the WT microglia. In FcγR^−/−^ microglia, there was still uptake of aggregated human α-SYN but the pattern of intracellular α-SYN was quite different, with diffuse labeling of small puncta throughout the cytoplasm. Scale bars: panel **A, B, E, F** bar=50 μm; panel **C, D, G, H** bar=20 μm. Image 1 in Panels **D** and **H** are confocal images of WT and FcγR^−/−^ mouse primary microglia treated with aggregated human α-SYN, respectively; 2 and 3 in Panel **D** and **H** are transverse and lengthwise images of z-stack series of the indicated cells. (**I**) 24 h after the treatment of aggregated human α-SYN, WT and FcγR^−/−^ mouse primary microglia were immunostained for human α-SYN (Green) and autophagosomal marker LC3B (Red). α-SYN co-localized with LC3B in WT but not FcγR^−/−^ microglia. Phase contrast images show the morphology of the cell. Scale bar=10 μm.

Internalization of aggregated α-SYN was studied 24, 48, and 72 h after the treatment of mouse primary microglia *in vitro*. Staining for α-SYN was performed using an anti-human α-SYN specific antibody, together with the microglial marker CD45. In microglia from wild-type animals, there was α-SYN internalization as early as 24 h, and we observed large dense α-SYN aggregates within the microglia at 72 h (Figure 1A-H). In general, most microglia contained a single dense aggregate. In microglia derived from FcγR^−/−^ animals, there was also uptake of the exogenous aggregated human α-SYN, but the pattern of intracellular localization was different. Rather than a single large aggregate, the intracellular α-SYN in the FcγR^−/−^ microglia was dispersed into a large number of smaller punctate areas of staining. In addition, the intensity of the staining for CD45 was reduced in both vehicle and α-SYN-treated FcγR^−/−^ primary microglia.

To identify the compartments containing α-SYN in the treated microglia, we performed double staining for α-SYN and LC3B, a marker for autophagosomes. At 24 h post treatment, we observed clear co-localization of α-SYN and LC3B within the aggregates in WT primary microglia but not in FcγR^−/−^ microglia (Figure 1I). This result suggests that in WT microglia, the internalized α-SYN is indeed targeted to autophagosomes, while in the FcγR^−/−^ microglia it appears to be trafficked to a set of distinct compartments.

### Aggregated human α-SYN triggers NF-κB activation with nuclear accumulation of p65 protein in microglia

**Figure 2:**
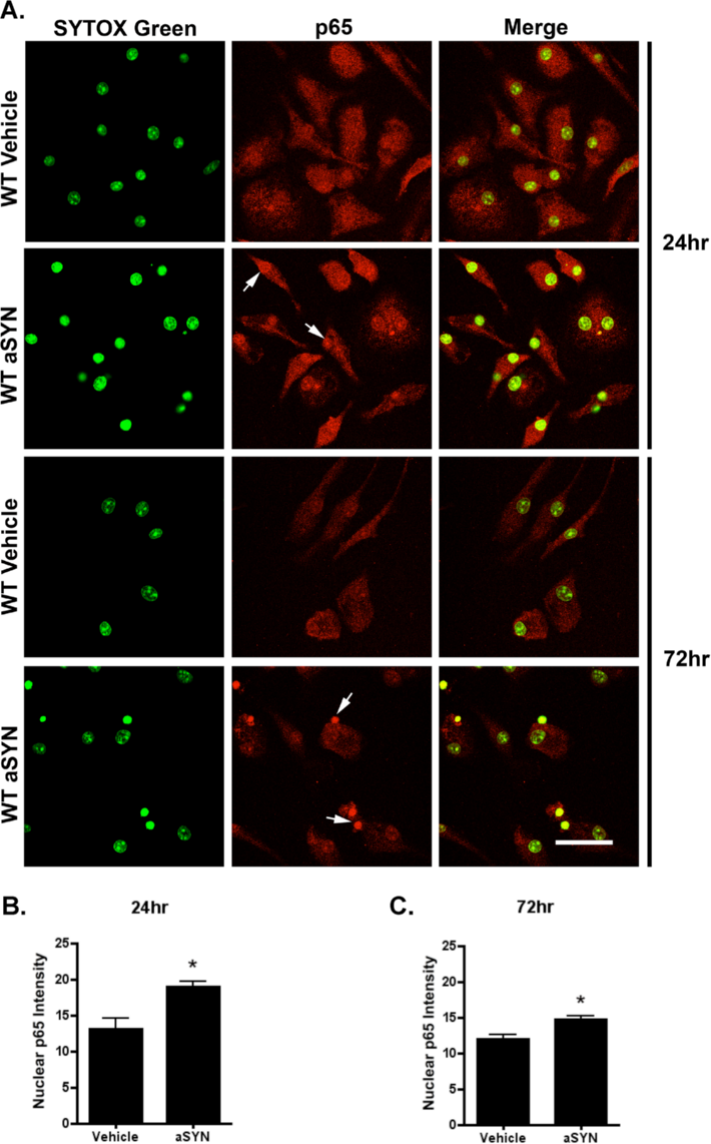
α**-SYN-induced NF-κB activation in WT mouse primary microglia.** (**A**) 24 h and 72 h after the treatment of either vehicle or 500 nM aggregated human α-SYN, cells were stained for NF-κB p65 protein (Red) and SYTOX Green to show the nucleus. At both time points, α-SYN-treated microglia exhibited increased immunoreactivity for NF-κB p65, and the nuclear accumulation of NF-κB p65 was quite distinct compared with the vehicle treated ones. Scale bar=40 μm. Arrows indicate the enrichment of nuclear NF-κB p65. (**B, C**) Quantification of 24 h and 72 h nuclear NF-κB p65 intensity. At least four images from each group were analyzed using ImageJ software. At both time points α-SYN-treated WT microglia had markedly enhanced nuclear NF-κB p65 staining compared with the vehicle-treated controls. *P<0.05, α-SYN *vs.* vehicle, *t*-test.

WT mouse primary microglia were treated with either vehicle or 500 nM aggregated human α-SYN for 24 h and 72 h, and immunocytochemistry for NF-κB p65 protein was performed to evaluate NF-κB activation *in vitro.* The cells were stained with SYTOX Green to show the nucleus. Both at 24 h and 72 h, α-SYN-treated microglia exhibited increased immunoreactivity for NF-κB p65, and the nuclear accumulation of NF-κB p65 was quite distinct compared with the vehicle treated cells (Figure 2A). In order to quantify the intensity of nuclear NF-κB p65, at least four images from each group were analyzed using ImageJ software. The nucleus was circled for ROI selection in the SYTOX Green/NF-κB p65 double staining images, and the nuclear NF-κB p65 intensity was obtained under the NF-κB p65 single channel images. This analysis confirmed the impression of markedly enhanced nuclear NF-κB p65 staining in the α-SYN-treated WT microglia compared to the vehicle-treated controls (Figure 2B and C).

### FcγR^−/−^ blocks α-SYN-induced nuclear NF-κB p65 accumulation in microglia

**Figure 3:**
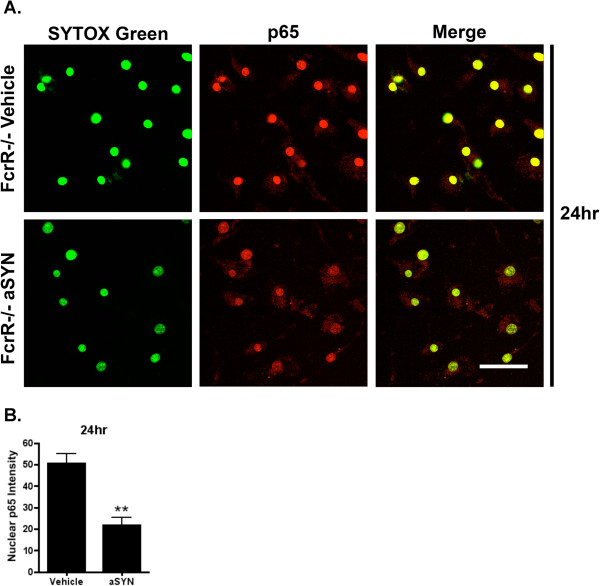
**α-SYN-induced NF-κB activation was blocked in FcγR**^**−/−**^**mouse primary microglia.** (**A**) 24 h after treatment with either vehicle or 500 nM aggregated human α-SYN, FcγR^−/−^ microglia were stained for NF-κB p65 protein (Red) and SYTOX Green. α-SYN-treated microglia exhibited attenuated immunoreactivity for NF-κB p65 compared with the vehicle-treated ones. Scale bar=40 μm. (**B**) Quantification of 24 h nuclear NF-κB p65 intensity. After the treatment of α-SYN, FcγR^−/−^ microglia had a significant decrease in nuclear p65. **P<0.01, α-SYN *vs.* vehicle, *t*-test.

We performed the same SYTOX Green/NF-κB p65 double staining for vehicle and α-SYN-treated FcγR^−/−^ microglia 24 h and 72 h post treatment, and did the same quantification using ImageJ. Compared with WT microglia, vehicle-treated FcγR^−/−^ microglia showed a striking increase in nuclear p65 at baseline with intense staining of the nucleus at 24 h (Figure 3A), and this effect was not seen at 72 h (data not shown). This is of interest because it parallels the striking increase in nuclear p65 we have previously reported in the FcγR^−/−^ mice *in vivo*[15]*.* After treatment of FcγR^−/−^ microglia with aggregated α-SYN, the nuclear prominence of p65 staining was still evident, but quantification of the intensities demonstrated that the nuclear p65 decreased, rather than increased, 24 h after α-SYN treatment (Figure 3B) and there was no significant difference in staining intensity at 72 h (data not shown).

### FcγR^−/−^ attenuates α-SYN-induced NF-κB signaling and downstream expression of pro-inflammatory molecules

In order to investigate the effect of Fc gamma chain deletion on the NF-κB signaling and the production of cytokines/chemokines regulated by NF-κB, we performed ELISA and multiplexed assay on the conditioned media collected from the vehicle and α-SYN-treated primary microglia.

**Figure 4:**
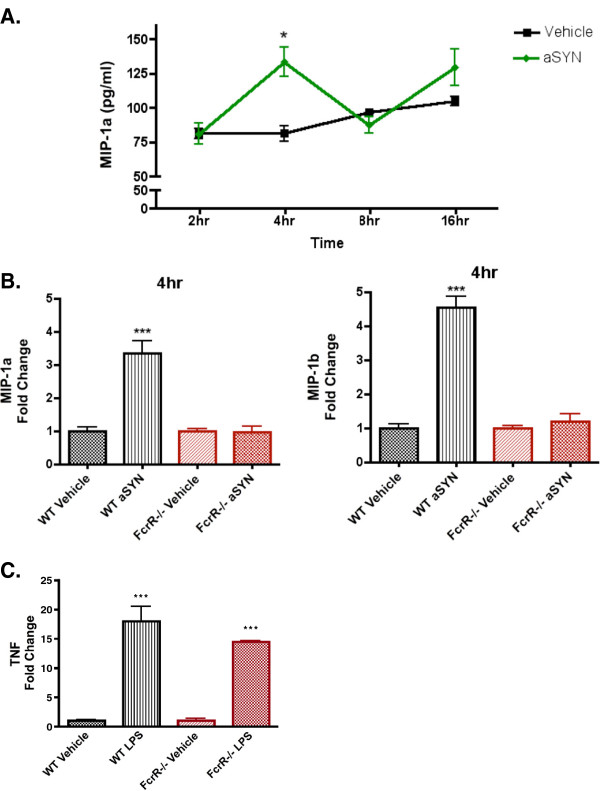
**FcγR**^**−/−**^**attenuated α-SYN-induced NF-κB signaling and downstream expression of pro-inflammatory molecules.** (**A**) MIP-1α ELISA on the conditioned media collected 2, 4, 8, and 16 h from the vehicle and α-SYN-treated WT primary microglia. At 4 h, but not the other time points, the MIP-1α level was significantly increased with α-SYN treatment compared with the vehicle controls. *P<0.05, α-SYN *vs.* vehicle, *t*-test. (**B**) Conditioned media were collected 4 h and 24 h after the treatment of vehicle or aggregated human α-SYN on WT and FcγR^−/−^ microglia for a 25-plex mouse cytokine/chemokine assay. At 4 h, MIP-1α and MIP-1β were significantly increased with α-SYN treatment in WT microglia but not FcγR^−/−^ microglia compared with vehicle-treated ones. All chemokine expression levels were normalized to the level of vehicle-treated WT or FcγR^−/−^ microglia, respectively. ***P <0.001 WT α-SYN *vs.* WT vehicle. One-way ANOVA with Tukey’s multiple comparison test. (**C**) Conditioned media collected from primary WT and FcγR^−/−^ microglia treated overnight with 100 ng/mL LPS. Both WT and FcγR^−/−^ microglia respond normally to TLR4 stimulation. TNF expression levels were normalized to the level of vehicle-treated WT or FcγR^−/−^ microglia, respectively. ***P <0.001 WT LPS *vs.* WT vehicle, FcγR^−/−^ LPS *vs.* FcγR^−/−^ vehicle. One-way ANOVA with Tukey’s multiple comparison test.

MIP-1α (Macrophage inflammatory protein-1α) is a marker for microglial activation and a target gene regulated by NF-κB [19], therefore we used it to characterize the time course of the effect of aggregated human α-SYN on pro-inflammatory molecules downstream of NF-κB. Conditioned media were collected 2, 4, 8, and 16 h post α-SYN treatment of WT microglia and were analyzed for MIP-1α ELISA. The MIP-1α level peaked at 4 h after the human α-SYN treatment (Figure 4A). Since α-SYN triggered nuclear p65 enrichment in WT microglia but not FcγR^−/−^ microglia at 24 h, we chose 4 h and 24 h as the time points for multiplex assay.

Conditioned media were collected 4 h and 24 h after the treatment of WT and FcγR^−/−^ microglia with either vehicle or aggregated human α-SYN, and studied using a 25-plex mouse cytokine/chemokine assay. To our surprise, there was only a limited cytokine response, with detectable levels of IL-1α, IP-10, MIP-1α, MIP-1β, MIP-2, MCP-1 at 4 h and 24 h. Other pro-inflammatory cytokines such as TNF-α or IL-6 were not detectable (< 10 pg/mL) in the multiplex assay (Table 1). At 4 h, MIP-1α and MIP-1β were significantly increased with α-SYN treatment in WT microglia compared with vehicle-treated cells, nevertheless, this increase was not observed in α-SYN-treated FcγR^−/−^ microglia (Figure 4B). To determine if FcγR^−/−^ microglia can respond normally to pathogen associated molecular patterns (PAMPs) involved in the NF-κB-dependent pro-inflammatory response, primary WT and FcγR^−/−^ microglia were plated and treated overnight with 100 ng/mL lipopolysaccharide (LPS), a Toll-like receptor 4 (TLR4) specific ligand. Conditioned media were collected and analyzed for TNF expression by ELISA. Following LPS treatment, we found no difference in amount of TLR4-specific TNF induction between the WT and FcγR^−/−^ microglia (Figure 4C) indicating that FcγR^−/−^ microglia can respond normally to pro-inflammatory stimuli.

**Table 1:** Effects of FcγR−/− on α-SYN-induced pro-inflammatory molecules

**Fold**	**4 h**				**24 h**			
	**WT vehicle**	**WT α-SYN**	**FcγR**^**−/−**^**vehicle**	**FcγR**^**−/−**^**α-SYN**	**WT vehicle**	**WT α-SYN**	**FcγR**^**−/−**^**vehicle**	**FcγR**^**−/−**^**α-SYN**
IL-1α	1.00±0.48	3.32±2.45	1.00±0.21	0.55±0.22	1.00±0.17	1.05±0.23	1.00±0.20	1.03±0.42
IP-10	1.00±0.21	5.29±2.93	1.00±0.41	0.79±0.38	1.00±0.23	1.01±0.34	1.00±0.07	1.79±0.42
MIP-1α	1.00±0.21	3.35±0.61^a^	1.00±0.11	0.97±0.39	1.00±0.09	1.29±0.35	1.00±0.31	2.07±0.93
MIP-1β	1.00±0.23	4.54±0.56^a^	1.00±0.15	1.19±0.45	1.00±0.08	1.26±0.38	1.00±0.46	2.91±1.55
MIP-2		nd			1.00±0.20	1.37±0.21	1.00±0.46	1.40±0.75
MCP-1		nd			1.00±0.12	1.24±0.37	1.00±0.31	1.88±1.10

Conditioned media were collected 4 h and 24 h after the treatment of vehicle or aggregated human α-SYN on WT and FcγR^−/−^ microglia for a 25-plex mouse cytokine/chemokine assay. At 4 h, MIP-1α and MIP-1β were significantly increased with α-SYN treatment in WT microglia but not FcγR^−/−^ microglia compared with vehicle-treated ones. All cytokine and chemokine expression levels were normalized to the level of vehicle-treated WT or FcγR^−/−^ microglia, respectively.

^a^*P* <0.001 WT α-SYN *vs.* WT vehicle. ANOVA with Tukey’s multiple comparison test. nd, not detectable.

## Discussion

Our studies have shown that aggregated α-SYN can interact directly with microglia, and can be internalized and condensed within these cells. With α-SYN treatment, there is enrichment of the NF-κB component p65 in the nucleus of microglia. Meanwhile, downstream chemokines regulated by NF-κB including MIP-1α and MIP-1β show increased expression levels. Deficiency of gamma chain subunit of the Fc receptors alters the pattern of internalized α-SYN so that it is no longer condensed in autophagosomes. It prevents microglial nuclear p65 accumulation, and blocks α-SYN-induced changes in chemokine expression.

In the mouse, the classic FcγRs are well characterized and include FcγRI, FcγRIIB, and FcγRIII. Both FcγRI and FcγRIII are multi-chain complexes composed of a single ligand-binding α-chain and a homodimer of common gamma-chains that mediates intracellular signaling through an immuno-receptor tyrosine-based activation motif (ITAM) in the cytoplasmic domain [20]. The FcγR^−/−^ mice that we used in our studies are deficient in the gamma chain subunit of the Fc receptors, therefore the functional expression of FcγRI and FcγRIII is greatly diminished, and the activated FcγR^−/−^ microglia lack the ability to phagocytose antibody-coated particles even with the retention of FcγRIIB [16]. Thus, although our data clearly implicate receptors containing the Fc gamma chain, they do not allow us to distinguish between effects mediated by FcγRI or FcγRIII, and it is possible that either, both, or additional scavenger receptors are involved in the α-SYN-induced neuroinflammation.

We have previously characterized the responses to α-SYN *in vivo* using an AAV-synuclein mouse model of PD [15]. We found that with the targeted overexpression of human α-SYN in the SN of WT mice, there is microglial activation and marked accumulation of p65 protein in the nucleus of microglia, and downstream activation of NF-κB-driven pro-inflammatory mediators can be detected. In FcγR^−/−^ mice, microglial nuclear p65 accumulation and transcriptional induction of the pro-inflammatory mediators in response to overexpression of α-SYN are blocked *in vivo*. Moreover, α-SYN-trigged dopaminergic neurodegeneration is attenuated. Our *in vitro* studies are consistent with these *in vivo* results, showing that excess α-SYN leads to microglial NF-κB activation and downstream pro-inflammatory signaling, and the gamma chain subunit of the Fc receptors is essential for this process. We also have observed both *in vivo* and *in vitro* that the FcγR^−/−^ mice have much greater baseline abundance of nuclear p65 (Figure 3). Both *in vivo* and *in vitro*, we have found that α-SYN induces a marked enhancement of nuclear p65 and activates NF-κB signaling, while in the absence of Fc gamma chain, α-SYN leads to a modest reduction in nuclear p65 (which is elevated at baseline) and does not trigger expression of NF-κB-dependent transcripts.

Using the *in vitro* approach, we have been able to investigate directly the interaction between aggregated α-SYN and microglia. We have found that α-SYN is internalized by microglia and concentrated in autophagosomes. As this α-SYN internalization occurs in the absence of any antibody mediation, this must represent a form of IgG-independent phagocytosis. The process of IgG-independent phagocytosis plays a critical role in the early response to infection, and is an important part of the innate immune system [21]. IgG-independent phagocytosis can be triggered by several different molecules expressed on the cell surface, such as scavenger receptors and complement receptors [21,22]. IgG-independent phagocytosis has been previously reported with α-SYN [23], and can also be mediated by FcγRs through interaction with alternative ligands including complement receptors and c-reactive proteins [12-14]. A recent study in Alzheimer’s disease showed that complement receptor type 3 (CR3), which is a receptor for soluble FcγRIII, contributes to the phagocytosis and clearance of fibrillar Aβ by microglia, and the internalized Aβ is transported to lysosomes in microglia [12,24].

Internalization of α-SYN was observed in both WT and FcγR^−/−^ microglia, but the intracellular destinations of the internalized protein were different. In WT microglia, α-SYN is trafficked to autophagosomes, as demonstrated by the co-localization with LC3. This is consistent with earlier work showing autophagocytic protein is localized in Lewy bodies [25], and that the engagement of FcγRs during phagocytosis induces recruitment of the autophagy protein LC3 to phagosomes [26]. In the FcγR^−/−^ microglia with deficiency of the gamma chain subunit of Fc receptors, however, the pattern of α-SYN internalization is altered, with a much more diffuse localization. In an effort to identify the compartments with α-SYN staining in the FcγR^−/−^ microglia, we performed double staining for α-SYN and lysosomal marker LAMP-1 but did not observe evidence for co-localization in either WT or FcγR^−/−^ microglia (data not shown). While the location of α-SYN in the FcγR^−/−^ microglia is uncertain, it is possible that it still involves Fc receptors. Deletion of the gamma chain results in complete loss of FcγRIII function and marked reduction of FcγRI, while FcγRII receptors are unaffected. There are previous studies suggesting that FcγRII may target proteins to recycling pathways, and it is possible that a similar process is at work in the gamma chain deficient microglia [27].

Although we did observe a direct effect of aggregated α-SYN on NF-κB-mediated chemokine expression, the extent of this response was limited and pro-inflammatory cytokines like TNF-α or IL-6 were not detectable at both 4 h and 24 h in the multiplex assay. This differs somewhat from prior studies in which a broader cytokine and chemokine response has been observed *in vitro*. These differences may arise in part because of the antigen employed; most prior studies used with mutant or nitrated α-SYN [28,29], or very high concentrations of wild-type α-SYN (up to 10 μM [30]). On the other hand, in our *in vivo* studies in the AAV-synuclein mouse model of PD, we did find a broad pattern of cytokine induction, with significant increases in the expression level of TNF-α, IL-6, and IL-1α [11]. The differences between the *in vitro* and *in vivo* responses point to the possibility that the microglial response may not be entirely cell-autonomous, and may require interactions with other cell types including T cell populations, which are known to be present in the brain in both the AAV model of PD as well as in the human disease [5,11].

## Conclusions

In summary, our data provide evidence that α-SYN can interact directly with microglia to induce pro-inflammatory signaling, and FcγR proteins mediate α-SYN intracellular trafficking and pro-inflammatory signaling. Therefore, inhibition of either FcγR signaling or downstream NF-κB activation may be viable therapeutic strategies to slow or prevent the progression of human PD.

## Abbreviations

α-SYN: Alpha-synuclein; AAV: Adeno-associated virus; DA: Dopamine; FcγR: Fc gamma receptor; IgG: Immunoglobulin G; LPS: Lipopolysaccharide; NF-κB: Nuclear factor kappa-light-chain-enhancer of activated B cells; PAMPs: Pathogen associated molecular patterns; PD: Parkinson’s disease; SNpc: Substantia nigra pars compacta.

## Competing interests

The authors declare that they have no competing interests.

## Authors’ contributions

SC carried out ELISA, multiplex assay and data analysis, participated in the design of the study, primary microglia culture and immunocytochemistry, and drafted the manuscript. DS participated in the design of the study and helped to draft the manuscript. AH participated in the design of the study, primary microglia culture and immunocytochemistry, and helped to draft the manuscript. All authors read and approved the final manuscript.

## Supplementary Material

Additional file 1 **Figure S1.** α-SYN preparation and aggregation. (**A**) Human α-SYN recombinant protein was purchased and resuspended at a concentration of 1 mg/mL and aggregated by heat and agitation for 1 week. α-SYN fractions were separated on Superdex columns and analyzed by western. Fractions 6 to 10 were combined and concentrated. Western analysis indicated aggregates of about 1 MDa. Click here for file
