# Supplementary figures and images for: The gamma chain subunit of Fc receptors is required for alpha-synuclein-induced pro-inflammatory signaling in microglia
Source: J Neuroinflammation. 2012 Nov 27;9:259. doi: 10.1186/1742-2094-9-259 (PMC3526448; doi:10.1186/1742-2094-9-259)

Supplemental 1.

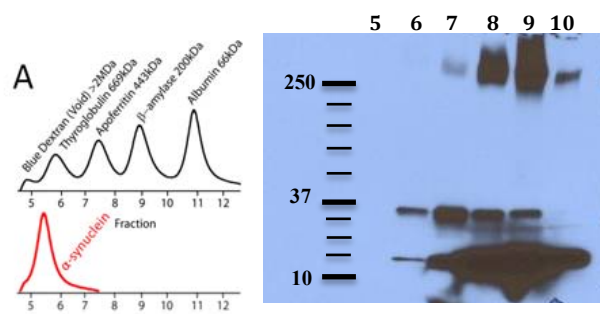

Supplement: Additional file 1 — Figure S1. α-SYN preparation and aggregation. (A) Human α-SYN recombinant protein was purchased and resuspended at a concentration of 1 mg/mL and aggregated by heat and agitation for 1 week. α-SYN fractions were separated on Superdex columns and analyzed by western. Fractions 6 to 10 were combined and concentrated. Western analysis indicated aggregates of about 1 MDa. [file 1742-2094-9-259-S1.pdf]
